# Surgical Outcomes and EEG Prognostic Factors After Stereotactic Laser Amygdalohippocampectomy for Mesial Temporal Lobe Epilepsy

**DOI:** 10.3389/fneur.2021.654668

**Published:** 2021-05-17

**Authors:** Shasha Wu, Naoum P. Issa, Maureen Lacy, David Satzer, Sandra L. Rose, Carina W. Yang, John M. Collins, Xi Liu, Taixin Sun, Vernon L. Towle, Douglas R. Nordli, Peter C. Warnke, James X. Tao

**Affiliations:** ^1^Department of Neurology, The University of Chicago, Chicago, IL, United States; ^2^Department of Psychiatry, The University of Chicago, Chicago, IL, United States; ^3^Department of Neurosurgery, The University of Chicago, Chicago, IL, United States; ^4^Department of Radiology, The University of Chicago, Chicago, IL, United States; ^5^Department of Neurology, Wuhan University, Wuhan, China; ^6^Department of Neurology, Beijing Electric Power Hospital, Beijing, China; ^7^Department of Pediatric Neurology, The University of Chicago, Chicago, IL, United States

**Keywords:** epilepsy surgery, medication resistant epilepsy, anterior temporal lobectomy, laser ablation, selective amygdalohippocampectomy, minimally invasive epilepsy surgery

## Abstract

**Objective:** To assess the seizure outcomes of stereotactic laser amygdalohippocampectomy (SLAH) in consecutive patients with mesial temporal lobe epilepsy (mTLE) in a single center and identify scalp EEG and imaging factors in the presurgical evaluation that correlate with post-surgical seizure recurrence.

**Methods:** We retrospectively reviewed the medical and EEG records of 30 patients with drug-resistant mTLE who underwent SLAH and had at least 1 year of follow-up. Surgical outcomes were classified using the Engel scale. Univariate hazard ratios were used to evaluate the risk factors associated with seizure recurrence after SLAH.

**Results:** The overall Engel class I outcome after SLAH was 13/30 (43%), with a mean postoperative follow-up of 48.9 ± 17.6 months. Scalp EEG findings of interictal regional slow activity (IRSA) on the side of surgery (*HR* = 4.05, *p* = 0.005) and non-lateralizing or contra-lateralizing seizure onset (*HR* = 4.31, *p* = 0.006) were negatively correlated with postsurgical seizure freedom. Scalp EEG with either one of the above features strongly predicted seizure recurrence after surgery (HR = 7.13, *p* < 0.001) with 100% sensitivity and 71% specificity.

**Significance:** Understanding the factors associated with good or poor surgical outcomes can help choose the best candidates for SLAH. Of the variables assessed, scalp EEG findings were the most clearly associated with seizure outcomes after SLAH.

## Introduction

Temporal lobe epilepsy (TLE) is the most common type of medication-resistant epilepsy. Despite the development of new generations of anti-seizure medications (ASMs), 30–40% of patients become resistant to ASM treatment (1). Anterior temporal lobectomy (ATL) and selective amygdalohippocampectomy (SAH) have been the gold standard surgical interventions for these patients, achieving 60–80% seizure freedom, though in highly selected patient cohorts (2–5). However, patients are often hesitant or unwilling to consider open surgery due to the fear of associated morbidity of a craniotomy, the concern for potential neurological deficits, and the risk of significant cognitive decline (6).

In recent years, there has been a shift from open resection to minimally invasive epilepsy surgery to minimize the complications associated with craniotomy and resection. Emerging data have shown that stereotactic laser amygdalohippocampectomy (SLAH) performed using MR-guided laser interstitial thermal therapy (MRgLITT) is a safe and effective alternative to open surgery for patients with mesial temporal lobe epilepsy (mTLE) (7, 8). In a recent study of 234 patients from 11 epilepsy centers, 58% of patients who underwent SLAH achieved Engel I outcome after 1 and 2 years of postoperative follow-up (9). Compared to open resection, SLAH is more tolerable and offers superior neurocognitive outcomes by sparing the lateral temporal neocortex (10). SLAH has been adopted as the first-line surgical option in many epilepsy centers in the United States for patients with mTLE with or without mesial temporal sclerosis (MTS).

Although early data showed that the seizure freedom rate of SLAH is close to or slightly inferior to that of traditional open surgery (11, 12), its long-term seizure outcome has not been determined. Prognostic factors for identifying ideal candidates for SLAH in a not highly selected group have not been established. In this retrospective study, we aim to assess the seizure outcomes of SLAH in patients with at least 1 year of postoperative follow-up and to identify prognostic factors from the presurgical scalp EEG and imaging that influence the surgical outcomes.

## Materials and Methods

### Patients and History

Thirty consecutive patients with drug-resistant TLE who underwent SLAH by MRgLITT at the University of Chicago Medical Center from January 2014 to December 2019 with at least 1 year follow-up were included in the study. Patients with bilateral interictal activity or bilateral seizures with one side clearly predominating were also included making this cohort more heterogeneous than previously reported (11, 12). In patients who underwent a second SLAH due to persistent seizures, outcomes were reported with respect to reoperation. Exclusion criteria were prior open temporal lobe surgery, ablation of structures outside the mesial temporal lobe at the same time as SLAH, and postoperative psychogenic non-epileptic seizures that precluded Engel classification. Patients' charts, imaging and EEG data were retrospectively reviewed.

### Presurgical Evaluation

All patients underwent a comprehensive neurological history interview and examination, inpatient video-EEG monitoring, brain MRI with volumetric 1.0 mm-section T1 coronal and fluid-attenuated inversion recovery (FLAIR) images to assess hippocampal volume, 18-fluorodeoxyglucose positron emission tomography (FDG-PET) (except patients 1, 2, and 28), neuropsychological evaluation and functional MRI (fMRI). Scalp video EEG was recorded using Xltek, Natus Medical Incorporated (Pleasanton, California, USA) at a sampling rate of 512 Hz. Electrodes were placed based on the international 10–20 arrangement with supplementary sub-temporal electrodes F9, T9, F10, T10 from the 10–10 system, and mastoid electrodes M1, M2. Scalp EEG filter settings are 1–70 Hz with notch filter on. No automated artifact rejection was used.

Ictal and interictal electrographic patterns were tabulated. Temporal intermittent rhythmic delta activity (TIRDA) is defined as short bursts of repetitive, rhythmic, 1–4 Hz activity of 50–100 mV in amplitude, predominantly running over the anterior temporal regions (13). Interictal regional slow activity (IRSA) is defined as delta activity over the temporal region, either continuous and polymorphic or intermittent and rhythmic on the same side of surgery which is present for more than 50% of the recording (14). An ictal onset EEG pattern in which the side of onset cannot be determined in one or more seizures was categorized as a “non-lateralizing” seizure onset. Patient 7 had seizures recorded independently from both right and left temporal regions on scalp EEG and was categorized as “contra-lateralizing” seizure onset because a minority of seizures lateralized to the contralateral (non-operative) side.

Brain imaging patterns were classified for each subject. Mesial temporal sclerosis (MTS) was defined as the presence of MRI T_2_/FLAIR signal hyperintensity with reduced hippocampal volume or loss of hippocampal internal architecture (15). 18F-FDG PET area of hypometabolism was classified as ipsilateral if it was on the same side of seizure onset and subsequent SLAH, bilateral if hypometabolism was detected in both temporal regions, and multifocal if hypometabolism involved the ipsilateral or bilateral frontal, parietal, or occipital lobe in addition to the temporal lobe.

Intracranial EEG recording with depth (stereo-electroencephalography, SEEG) and/or subdural electrodes was performed in 24 of 30 patients. Intracranial recording was indicated when patients had a normal brain MRI, when there was concern for lateral temporal neocortical onset, extratemporal onset, or if bilateral temporal onset could not be ruled out during non-invasive evaluation. Simultaneous scalp and invasive EEG were recorded using the above-mentioned recording system at a sampling rate of 1,024 Hz. Intracranial EEG filter settings are 1–100 Hz with notch filter off. Eleven of 24 patients had bitemporal intracranial recording due to concerns for bi-mesial temporal onset. The methodology of intracranial recording was detailed in our previous publication (16).

### Stereotactic Laser Amygdalohippocampectomy (SLAH)

Patients were eligible for SLAH when their intracranial EEG onset localized to mesial temporal structures or predominantly from one mesial temporal lobe in patients with bilateral mesial temporal onset seizures (patient 7). SLAH was performed in all subjects using the Visualase system (Visualase Medtronic, MN USA) by the same neurosurgeon (PCW). The location and volume of the ablation were determined either based on the results of intracranial EEG monitoring (the seizure onset zone and immediate spreading zone were ablated) or, in the absence of intracranial electrophysiological data, with the intention of ablating the AHC. Typically, three to five lesions were generated along the longitudinal axis of AHC. The detailed surgical technique used for MRI-guided SLAH has been previously described (16). Seven of 30 patients had a second SLAH due to recurrent seizures (Table 1). The second SLAH was designed to target the residual AHC; the remnant mesial amygdala was the target of re-ablation in four of seven patients.

**Table 1:** Demographic information arranged by surgical outcomes.

**ID**	**Sex**	**Age**	**Epi duration**	**MTS**	**PET**	**Non TL EEG**	**IRSA**	**Non-lat or contra sz**	**Side**	**Postop (months)**	**Engel outcome**	**Time to sz**	**ASM reduction**	**2nd surg**
11	F	41	4	No	Yes	Yes	No	No	R	63	I	NA	No	No
22	M	56	55	No	No	No	No	No	R	44	I	NA	No	No
23	F	51	5	No	Yes	No	No	No	R	42	I	NA	Yes	No
26	F	22	5	No	Yes	No	No	No	R	37	I	NA	Yes	No
4	F	42	26	Yes	Yes	Yes	No	No	L	52	I	NA^*^	No	Yes
6	M	25	15	Yes	Yes	No	No	No	R	64	I	NA	Yes	No
9	M	42	41	Yes	Yes	No	No	No	L	28	I	NA^#^	No	Yes
10	F	46	30	Yes	No	No	No	No	R	66	I	NA	No	No
14	F	46	23	Yes	Yes	Yes	No	No	L	38	I	NA^@^	Yes	Yes
18	F	32	27	Yes	Yes	No	No	No	R	49	I	NA	Yes	No
20	F	61	34	Yes	Yes	Yes	No	No	L	39	I	NA^∧^	No	Yes
29	M	67	50	Yes	Yes	No	No	No	L	18	I	NA	No	No
31	M	69	3	Yes	Yes	No	No	No	L	12	I	NA	No	No
2	F	53	3	No	NA	No	Yes	No	R	78	II	17	Yes	No
15	F	58	11	Yes	No	Yes	Yes	No	R	59	II	10	No	No
17	M	32	23	Yes	Yes	Yes	Yes	No	L	50	II	24	Yes	No
3	M	65	57	Yes	No	Yes	No	Yes	L	74	II	18	Yes	No
19	M	32	22	No	No	No	No	No	R	48	II	18	Yes	No
25	F	46	45	Yes	Yes	Yes	No	No	R	39	II	12	Yes	No
21	F	50	43	No	No	Yes	Yes	No	L	45	III	2	Yes	No
12	M	29	25	Yes	Yes	Yes	Yes	No	L	51	III	2	No	Yes
13	M	32	11	No	Yes	No	No	Yes	L	60	III	1	No	No
24	F	60	13	No	No	Yes	No	Yes	R	42	III	3	No	No
5	F	36	7	No	Yes	Yes	No	No	L	66	III	1	No	No
8	F	50	6	No	Yes	No	No	No	R	69	III	1	No	No
30	F	21	6	Yes	Yes	Yes	No	No	R	14	III	8	No	No
16	F	41	33	No	No	Yes	Yes	Yes	R	41	IV	1	No	Yes
7	M	20	5	No	No	Yes	Yes	Yes	R	62	IV	3	No	Yes
28	M	63	1	Yes	NA	No	Yes	No	R	37	IV	1	No	No
1	M	20	17	No	NA	Yes	No	Yes	L	79	IV	1	No	No

*For patients who had a second surgery, time to seizure and postoperative follow-up duration are measured from the time of the second surgery*.

*Epi duration, duration of the epilepsy; MTS, ipsilateral mesial temporal lobe sclerosis; PET, ipsilateral temporal hypometabolism on positron emission tomography; non-TL EEG, non-temporal lobe interictal EEG findings; IRSA, interictal regional slow activity; Non-lat or contra sz, non-lateralizing or contra-lateralizing seizure onset; side, side of surgery; Post-op, post-op follow-up months; time to sz, time from surgery to first postoperative seizure; ASM; anti-seizure medications; 2nd surg, second surgery*.

* *Patient 4 had a seizure 11 months after the first ablation and was seizure free at 52 months after the second ablation*.

# *Patient 9 had a seizure 36 months after the first ablation and was seizure free at 28 months after the second ablation*.

@ *Patient 14 had a seizure 7 months after the first ablation and was seizure free at 38 months after the second ablation*.

∧ *Patient 20 had a seizure 1 month after the first ablation and was seizure free at 39 months after the second ablation*.

### Assessment of Seizure Outcomes

The current Engel class seizure status and the time from SLAH to the first seizure recurrence were utilized as the endpoints in the analysis. Engel classification is defined as the following: class I, free of seizures (patient may have aura); class II, rare disabling seizures; class III, worthwhile improvement; class IV, no worthwhile improvement (17). For patients who had a second SLAH, the postoperative outcome was based on the second SLAH if there were more than 1 year of follow-up after the second SLAH. Acute postoperative seizures that occurred in the first week after surgery were not counted as recurrent (18). Patients with seizures occurring after unsupervised ASM withdrawal (e.g., missed doses) with subsequent seizure-freedom for more than 2 years after resuming the medications were classified as having an Engel class I outcome with seizure freedom (17). Preoperative ASMs were maintained at least for 6 months after SLAH and in some cases were reduced thereafter if patients remained seizure-free or adjusted if the seizures were not controlled. The surgical complication rate from this cohort has been previously reported (16), and surgical complications are not considered endpoints as we assume that they occur sporadically.

### Data Analysis

The goal of the study was to establish prognostic factors in pre-surgical evaluation for the effect of SLAH on seizures. Parameters from the pre-surgical evaluation were tested as independent variables in a univariate Cox proportional hazard model with the outcome being the duration of postsurgical seizure freedom. The parameters tested were: (1) Unilateral MTS on MRI. (2) 18F-FDG PET showed unilateral temporal hypometabolism. (3) Presence of interictal regional slow activity (IRSA) ipsilateral to the side of surgery. (4) Presence of non-temporal interictal findings in the form of contralateral TIRDA, contralateral spikes or sharp waves, or extratemporal interictal epileptiform activity. (5) Presence of non- or contra-lateralizing seizure onset. Stata software was used for data analysis. A power analysis was performed to estimate effect size (Hazard Ratio) from the sample numbers available using a long-rank test (Freedman method; power = 0.8, alpha = 0.05) and an unbalanced design (the number of subjects with a particular characteristic was different from the number of subjects without that characteristic). A Bonferroni correction for multiple comparisons was made; an initial *p*-value of 0.05 was considered significant, which is reduced to 0.01 because five different parameters were tested.

## Results

### Demographic Data and Surgical Outcomes

Clinical data from 30 patients (17 female) with medically intractable TLE were reviewed and summarized in Table 1. The mean duration of epilepsy was 21.5 ± 16.9 years (mean ± standard deviation; range 1–57 years). The mean age at surgery was 43.6 ± 14.9 years (range 20–69 years). The mean postoperative follow-up was 48.9 ± 17.6 months (range 12–79 months).

At the most recent follow-up, 13/30 (43%) remained seizure-free after the most recent SLAH. The seizure freedom rate was 18/30 (60%) at 1 year and 3/10 (30%) at 5 years. The time from surgery to the first seizure varied between 1 and 36 months. In ten of 17 patients with seizure recurrence, seizures recurred within 6 months after surgery and all had Engel class III or IV outcomes. Seven patients with recurrent seizures underwent a second SLAH to ablate residual mesial temporal tissue (Table 1). Four of those seven were seizure-free for more than 1 year after the second SLAH. The remaining three continued experiencing seizures. Postoperative supervised reduction of at least one ASM was conducted in 11 of the 30 patients (Table 1).

### Factors Associated With Surgery Failure

Five parameters were assessed using a univariate Cox proportional hazards model to identify which, if any, associated with failure of surgery to produce seizure freedom. Hazard ratios >1.0 imply patients were likely to have recurrent seizures after surgery, and hazard ratios <1.0 imply patients were unlikely to have recurrent seizures after surgery. A power analysis suggests that an effect size of 2.7–3 (Hazard Ratio) would likely be detectable with 27–30 total subjects and the observed distribution of characteristics (unbalanced sampling). While several other parameters are likely associated with outcome, analysis was limited to five parameters because of the small overall sample size (30 subjects with 17 that had postoperative seizure recurrence). The calculated hazard ratios are listed in Table 2.

**Table 2:** Hazard ratios associated with characteristics identified during presurgical planning.

**Presurgical characteristic—univariate analysis**	**HR**	**p**	**95% Confidence interval**	**^#^with/without characteristic**	**^#^postop sz with/without characteristic**	**Months to 1st sz** **with/without characteristic**
No unilateral MTS	2.22	0.108	0.84–5.87	14/16	10/7	4.8/10.7
No unilateral PET hypometabolism	2.34	0.115	0.81–6.72	9/18	7/7	7.9/7.0
Non-temporal lobe interictal findings	2.80	0.056	0.97–8.03	16/14	12/5	7.1/7.6
Ipsilateral temporal lRSA	4.05	0.005	1.51–10.86	8/22	8/9	7.5/7.0
Non- or contra-lateralizing seizure onset	4.31	0.006	1.51–12.34	6/24	6/11	4.5/8.7
Non- or contra-lateralizing seizure onset or Ipsilateral temporal lRSA	7.13	<0.001	2.41–21.07	12/18	12/5	6.9/8.0

*Results of a univariate analysis, with each listed parameter tested as an independent variable. MTS, mesial temporal sclerosis on MRI. IRSA, interictal regional slow activity. HR, hazard ratio for recurrent seizures after surgery, calculated with the Cox proportional hazards model*.

# *with/without characteristic: the number of subjects in the cohort who had the defining characteristic/the number of subjects in the cohort who did not have the defining characteristic; there were 30 subjects in the cohort*.

# *postop sz with/without characteristic: the number of subjects in the group with the characteristic that had a postoperative seizure/the number of subjects in the cohort who did not have the defining characteristic that had a postoperative seizure. Months to 1st sz: the average number of months between surgery and the first seizure in subjects who had a seizure*.

The absence of unilateral MTS on MRI or of 18F-FDG PET hypometabolism restricted to the unilateral temporal appeared to be associated with a higher risk of seizure recurrence, but these relationships did not reach statistical significance with the small number of subjects available (Table 2). Three patients had MRI findings other than MTS. Patient 19 had diffuse band heterotopia in bifrontal, parietal and temporal regions. Patient 28 had ventricular enlargement due to normal pressure hydrocephalus. Patient 1 had right internal capsule gliosis.

Interictal scalp EEG patterns found outside of the surgical temporal lobe, consisting of bilateral or extratemporal sharp waves, or contralateral TIRDA, also appeared to be associated with a higher risk of seizure recurrence. However, this relationship did not reach statistical significance (Table 2). Sixteen of 30 patients had such interictal findings, and 12 of these 16 patients (75%) had postoperative seizures, on average 7.1 months after surgery. By comparison, only five of 14 patients (36%) with unilateral temporal lobe discharges had postoperative seizures, on average 7.6 months after surgery (HR 2.80, CI 0.97–8.03, *p* = 0.056).

**Figure 1:**
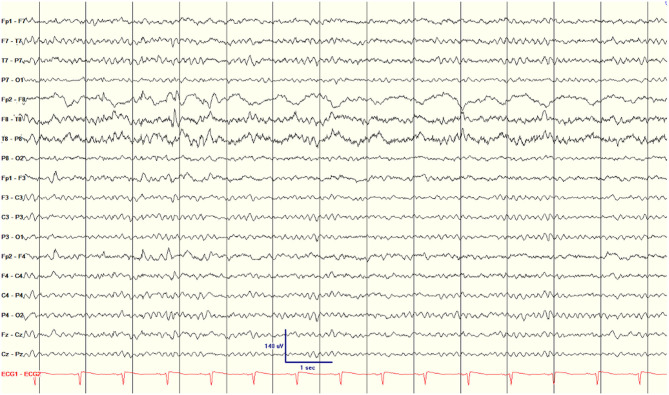
Scalp EEG showing nearly continuous interictal regional slow activity (IRSA) on the surgical side (right hemisphere) in patient 2.

One additional interictal scalp EEG parameter was found to be associated with seizure recurrence after surgery. All patients had focal slowing ipsilateral to the surgical side. Twenty-two patients had TIRDA and eight of 30 patients were noted to have prominent, near-continuous focal slowing over the surgical temporal lobe (Figure 1), similar to the interictal regional slow activity (IRSA) described by Koutroumanidis et al. (14) All eight patients with IRSA had recurrent seizures (100%, three Engel class II and five class III and IV), with an average of 7.5 months to first seizure after surgery. Koutroumanidis et al. (14) suggested that temporal IRSA was often associated with hypometabolism in the lateral posterior temporal lobe. A PET scan was available for six of the eight subjects with IRSA; in three there was hypometabolism in the lateral posterior temporal neocortex (patients 7, 12, and 15), with hypometabolism extending beyond the temporal cortex into the occipital cortex in two (patients 7 and 15). In the other three there was either no clear hypometabolism (patient 16), exclusively anterior temporal lobe hypometabolism (patient 17), or bilateral anterior temporal lobe hypometabolism (patient 21). For the 22 patients who did not have IRSA, only nine (41%) had seizures after surgery, on average 7.0 months after surgery. The resulting hazard ratio for IRSA was 4.05 (CI 1.51–10.86, *p* = 0.005, which is significant after Bonferroni correction for multiple comparisons; Table 2).

**Figure 2:**
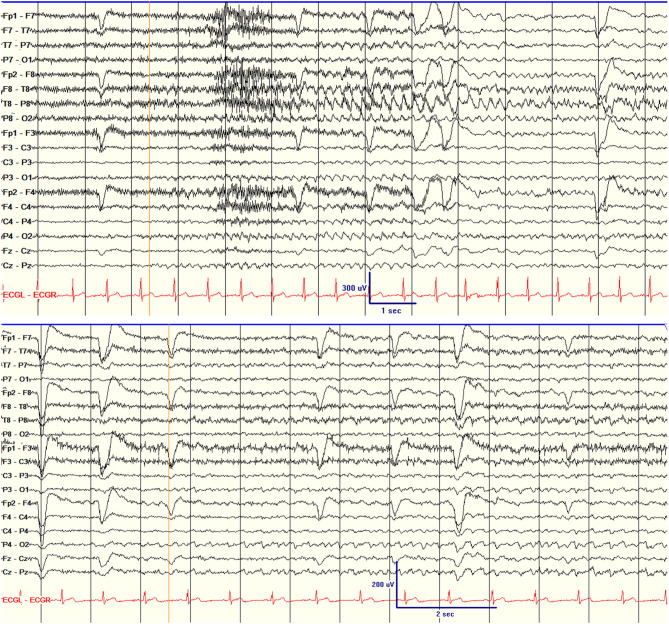
Scalp EEG showing the unilateral seizure onset (right temporal seizure onset) in patient 18 (top) and non-lateralizing seizure onset in patient 1 (bottom). The orange lines indicate EEG seizure onset.

Of the parameters assessed, ictal scalp EEG patterns had the closest association with surgery failure. Five patients had scalp ictal EEGs that were non-lateralizing for one or more seizures (Table 1; Figure 2). One of five had a class II outcome and the remaining four had class III or IV outcomes. Patient 7 had independent bilateral ictal onset on scalp EEG recording with most of the seizures starting over the right temporal region (R:L = 7:1). His intracranial recording showed independent bitemporal interictal epileptiform discharges (IEDs), but all seizures were recorded from the right hippocampus. The patient underwent right hippocampal SLAH and had a class IV outcome despite two SLAH on the right side. None of the six patients with either non-lateralizing or contra-lateralizing seizure onset were seizure-free (100% recurrence) compared to 11 of 24 (46%) seizure-recurrence in patients who had only unilateral seizure onset. The average time from surgery to first seizure was 4.5 months in patients with non- or contra-lateraling seizure onset compared to 8.7 months in patients with unilateral seizure onset (HR 4.31, CI 1.51–12.34, *p* = 0.006, which is significant after Bonferroni correction for multiple comparisons).

**Figure 3:**
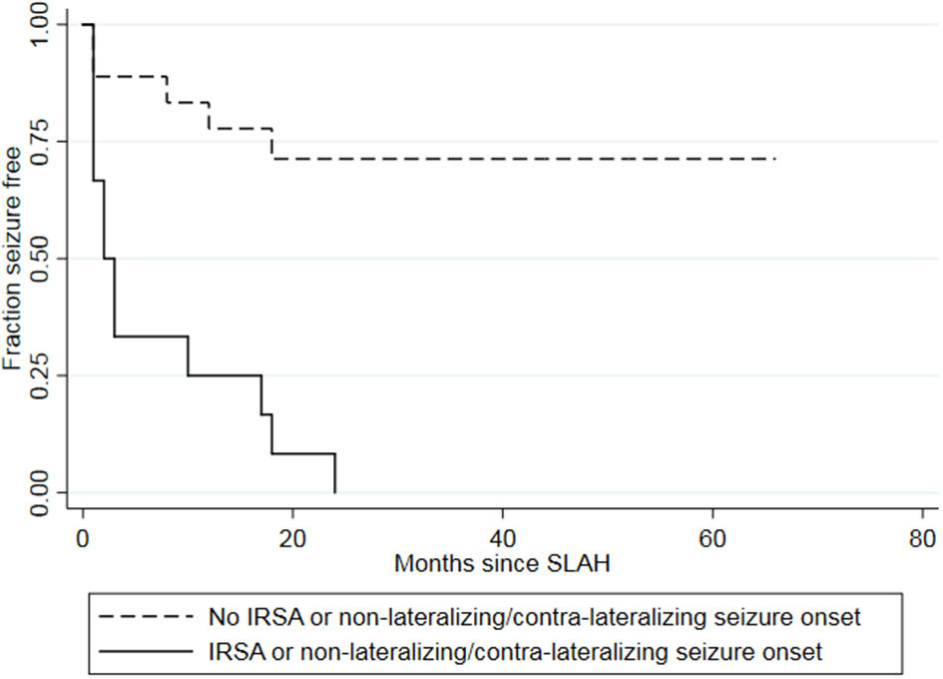
Kaplan-Meier survival curve showing longer seizure freedom for the group of patients without either IRSA or non- or contra-lateralizing seizure onset (dashed lines) than for the group of patients with either IRSA or non- or contra-lateralizing seizure onset (solid line). Two patients had both IRSA and a non- or contra-lateralizing seizure onset on EEG. IRSA, interictal regional slow activity; SLAH, stereotactic laser amygdalohippocampectomy.

Of the twelve patients who had either non- or contra-lateralizing seizure onset or ipsilateral IRSA, none were seizure-free after surgery (100%) (Table 2). The seizures occurred, on average, 6.9 months after surgery. By comparison, only five of 18 patients (28%) with neither of these EEG features had a postoperative seizure, with an average latency of 8 months. The presence of either IRSA or non- or contra-lateralizing ictal onset was associated with a hazard ratio of 7.13 (*p* < 0.001) for recurrent seizures, with 100% sensitivity and 71% specificity. The positive predictive value (PPV) was 0.72, with 13 of 18 patients without either of these patterns becoming seizure-free after surgery. The negative predictive value was 1.0, with all 12 patients with either IRSA or non- or contra-lateralizing ictal onset having a postsurgical seizure. A Kaplan-Meier survival curve shows the longer seizure freedom for the group of patients without either IRSA or non- or contra-lateralizing ictal onset compared to the group of patients with either EEG patterns (Figure 3).

## Discussion

Prognostic factors have been studied extensively for traditional ATL (19, 20). This study analyzed the association between presurgical characteristics and surgery outcome after SLAH in a cohort of patients that included patients with either unilateral or bilateral EEG findings. This approach was chosen following the findings that in lesional cases even bilateral EEG-changes did not preclude seizure freedom (21). A finding of either non- or contra-lateralizing ictal EEG onset or interictal ipsilateral temporal IRSA associated with the failure to achieve seizure freedom after SLAH with a sensitivity of 100% and a specificity of 71%.

Patients with bitemporal seizures are poor candidates for unilateral resection (22). However, the significance of non-lateralizing ictal EEG regarding surgery outcome is not known. We found that a non-lateralizing EEG or contra-lateralizing seizure onset for even a minority of captured seizures is significantly negatively associated with seizure outcome. The presence of a non-lateralizing ictal EEG could suggest fast bilateral synchronization or a contralateral seizure onset and lead to unfavorable surgical outcomes (23, 24).

Temporal intermittent focal slowing (TIRDA) is a well-known pattern that is considered an interictal marker for TLE (25, 26). By contrast, continuous focal or regional slowing (IRSA) usually suggests an underlying “structural” abnormality and is not considered epileptiform (27). In the current data set, however, IRSA on the surgical side was associated with recurrent seizures after SLAH (HR 4.05). The presence of IRSA could imply an extended epileptogenic zone beyond the mesial temporal region. This is supported by the imaging findings of Koutroumanidis et al. (14) in which IRSA was associated with hypometabolism in the lateral temporal neocortex. However, only half of the subjects that both IRSA and a PET scan showed lateral posterior temporal hypometabolism, suggesting that IRSA, even independent of PET findings, may be a negative predictor of surgical outcome. To our knowledge, this is the first description of IRSA in association with surgery outcomes in TLE.

Bi-temporal IEDs and postoperative contralateral TIRDA have been associated with poor surgical outcome while presurgical unilateral IEDs are good prognostic factors in surgery for TLE (28–30). Our findings suggested that interictal EEG findings outside the affected temporal lobe (bitemporal, extratemporal IEDs and contralateral TIRDA) were not significant independent factors to predict surgical outcome (HR 2.80, *p* = 0.06). However, based on a power analysis, the observed HR of 2.8 would be in the borderline detectable effect size for the small number of subjects (potential type I error), and a larger data set would be needed to determine whether this feature is a significant predictor of surgical failure.

Drug-resistant TLE associated with MTS has the best outcome after traditional ATL or SAH (31–33). Similar findings were also reported after SLAH from our previous study and other epilepsy centers (11, 12, 16, 34). Temporal PET hypometabolism has also been associated with a higher rate of seizure freedom after ATL (35). While the lack of unilateral MTS or ipsilateral PET appeared to be associated with a higher risk of seizure recurrence and a shorter time between surgery and first seizure, the relationships were not statistically significant during the long-term follow-up. Similar findings were reported from other groups recently (8, 36). Again, the observed hazard ratios were smaller than the detectable HR based on a power analysis (potential type I error), so a larger data set would clarify the relationship between the imaging parameters and postsurgical seizure freedom.

While the use of SLAH has been increasing, the long-term outcome after SLAH is not yet well-understood. We found that the seizure-freedom rate after SLAH decreases gradually during the first 5 years of postoperative follow-up in this cohort with bilateral EEG changes and additional IRSA. The long-term seizure outcome of SLAH appears to be marginally lower than that of ATL, in which complete seizure freedom was seen in 55.3% of patients at 2 years and in 47.7% at 5 years after surgery but the ATL cohorts are more selective (5). One possible reason for the difference in seizure freedom between ATL and SLAH could be due to incomplete ablation of extrahippocampal mesial tissues, as suggested by the recent study of Wu et al. (9) With SLAH, structures other than the amygdalohippocampal complex, including piriform, entorhinal, perirhinal, and parahippocampal cortices, are not typically targeted and are inconsistently ablated. These extra-hippocampal mesial structures can be involved in seizure generation and propagation in mTLE and are typically resected during ATL and SAH (37–40). Therefore, ablation of additional mesial temporal structures, perhaps using two laser probes targeting both longitudinal AHC and the mesial part of the amygdala, may be necessary to improve long-term seizure freedom. In a parallel study at our center, an analysis of ablated regions suggests that parahippocampal ablation is associated with seizure freedom in mesial temporal lobe epilepsy.

Another important factor in explaining the inferior seizure outcome after SLAH is the patient's preference. Because of the low risk of complications and much faster recovery time, almost all patients chose minimally invasive SLAH over traditional ATL or SAH when we offered these two options to treat temporal lobe epilepsy. In some patients, SLAH was offered as a “palliative” procedure due to significant seizure burden and comorbidity despite the presurgical evaluation suggesting the seizure focus was more extensive than a unilateral mesial temporal region. For example, palliative surgery without expectation for seizure freedom was offered to patient 7, who had bitemporal onset seizures on scalp EEG, and patient 19 who had double cortex on MRI brain.

## Study Limitations

There are two major limitations to this study. First, its single-center retrospective design limits generalizability since the patient population and indications for surgery may differ at other surgical centers. The more significant limitation is the small sample size. There are many other potential predictors of surgical outcome, for example the duration of epilepsy, seizure type, presence of non-MTS lesions, neuropsychological findings, the extent of hypometabolism, but with only 30 subjects it is not statistically appropriate to test all possibilities. We therefore limited our analysis to five parameters related to imaging and scalp EEG. However, to allow subsequent meta-analyses of other parameters, we have included additional data in a Supplementary Table. Similarly, the power analysis suggests that only large effects could be detected with 30 subjects (Hazard Ratios of ~3). With 17 events, defined as recurrent seizures after completed ablation, a multivariate analysis was not possible. This study is therefore preliminary, identifying two parameters that related to surgical failure with SLAH that should inform future larger prospective studies.

## Conclusion

In conclusion, of the variables assessed, scalp EEG findings were the most clearly associated with seizure outcomes after SLAH. Interictal regional slow activity and a non- or contra-lateralizing seizure onset are strong negative markers of prognosis after SLAH. The seizure-freedom rate after SLAH gradually decreases over the course of the initial 5-year postoperative follow-up. Understanding the factors associated with good or poor surgical outcomes can help the selection of the best candidates for SLAH and help predict the outcome before surgery. Multi-center and long-term follow-up studies are warranted to clarify the long-term safety and efficacy of SLAH for patients with mTLE.

## Data Availability Statement

The original contributions presented in the study are included in the article/Supplementary Material, further inquiries can be directed to the corresponding author.

## Ethics Statement

The studies involving human participants were reviewed and approved by The University of Chicago Biological Sciences Division/University of Chicago Medical Center AURA Institutional Review Board. The patients/participants provided their written informed consent to participate in this study.

## Author Contributions

SW and NI contributed to the study design, data collection, and manuscript preparation. XL and TS contributed to the data collection. PW performed the surgeries. DS, ML, SR, CY, JC, VT, DN, and JT contributed to the study design and data collection. All authors approved the final manuscript.

## Conflict of Interest

SW, NI, ML, SR, JC, PW, and JT are investigators in the Stereotactic Laser Ablation for Temporal Lobe Epilepsy (SLATE) trial funded by Medtronic. The remaining authors declare that the research was conducted in the absence of any commercial or financial relationships that could be construed as a potential conflict of interest.
