# Supplementary material for: Surgical Outcomes and EEG Prognostic Factors After Stereotactic Laser Amygdalohippocampectomy for Mesial Temporal Lobe Epilepsy
Source: Front Neurol. 2021 May 17;12:654668. doi: 10.3389/fneur.2021.654668 (PMC8165234; doi:10.3389/fneur.2021.654668)
Supplement: Supplementary file 1 [file Table_1.DOCX]

| **ID** | **Duration of epilepsy yr)** | **Seizure Type** | **Lateralizing semiology** |
| --- | --- | --- | --- |
| 1 | 17 | FIAS with rare BTC | None (automatism) |
| 2 | 3 | FIAS with rare BTC | None (automatism) |
| 3 | 57 | FIAS with rare BTC | None (automatism) |
| 4 | 26 | FIAS with rare BTC | None (automatism) |
| 5 | 7 | FIAS | None (behavior arrest) |
| 6 | 15 | FIAS with rare BTC | None (automatism) |
| 7 | 5 | BTC | None (behavior arrest) |
| 8 | 6 | FIAS with rare BTC | None (automatism) |
| 9 | 41 | BTC | None (behavior arrest) |
| 10 | 30 | FIAS with rare BTC | Right hemisphere (left hand dystonia) |
| 11 | 4 | FIAS with rare BTC | None (automatism) |
| 12 | 25 | BTC | None (automatism) |
| 13 | 11 | BTC | None |
| 14 | 23 | FIAS with rare BTC | None |
| 15 | 11 | FIAS with rare BTC | None (automatism) |
| 16 | 33 | FIAS with rare BTC | None |
| 17 | 23 | BTC | None |
| 18 | 27 | FIAS with rare BTC | Right (left hand dystonia) |
| 19 | 22 | BTC | None |
| 20 | 34 | FIAS with rare BTC | Left (right arm clonic movement) |
| 21 | 43 | BTC | None (automatism) |
| 22 | 55 | FIAS with rare BTC | Left version |
| 23 | 5 | FIAS | None |
| 24 | 13 | FIAS with rare BTC | Left version |
| 25 | 45 | FAS with rare BTC | Right (automatism with  preserved awareness and postictal nose wiping) |
| 26 | 5 | FIAS with rare BTC | Right (left version, left arm extension) |
| 28 | 1 | BTC | None |
| 29 | 50 | BTC | None (automatism) |
| 30 | 6 | BTC | None |
| 31 | 3 | FIAS | Left (right hand tonic) |

yr: year. FIAS: focal impaired awareness seizure. FAS: focal aware seizure. BTC: bilateral tonic-clonic seizure.
